# Bisphenol A Alters the Levels of miRNAs That Directly and/or Indirectly Target Neuropeptide Y in Murine Hypothalamic Neurons

**DOI:** 10.3390/genes14091773

**Published:** 2023-09-08

**Authors:** Kimberly W. Y. Mak, Wenyuan He, Neruja Loganathan, Denise D. Belsham

**Affiliations:** 1 Department of Physiology, University of Toronto, Medical Sciences Building 3247A, 1 Kings College Circle, Toronto, ON M5S 1A8, Canada; kimberly.mak@mail.utoronto.ca (K.W.Y.M.); wenyuan.he@mail.utoronto.ca (W.H.); neruja.loganathan@mail.utoronto.ca (N.L.); 2 Department of Medicine, University of Toronto, Toronto, ON M5S 1A8, Canada

**Keywords:** microRNAs, hypothalamus, neuropeptide Y, bisphenol A, energy homeostasis

## Abstract

The hypothalamus is a vital regulator of energy homeostasis. Orexigenic neuropeptide Y (NPY) neurons within the hypothalamus can stimulate feeding and suppress energy expenditure, and dysregulation of these neurons may contribute to obesity. We previously reported that bisphenol A (BPA), an endocrine disruptor with obesogenic properties, alters *Npy* transcription in hypothalamic neurons by inducing oxidative stress. We hypothesized that hypothalamic microRNAs (miRNAs), a class of small non-coding RNAs, could directly regulate *Npy* gene expression by binding the 3′ untranslated region (UTR). Five predicted *Npy*-targeting miRNA candidates were uncovered through TargetScan and were detected in *Npy*-expressing hypothalamic neuronal cell models and hypothalamic neuronal primary cultures. BPA dysregulated the expression of a number of these hypothalamic miRNAs. We examined the effects of putative *Npy*-targeting miRNAs using miRNA mimics, and we found that miR-143-3p, miR-140-5p, miR-29b-1-5p, and let-7b-3p altered *Npy* expression in the murine hypothalamic cell lines. Importantly, miR-143-3p targets the mouse *Npy* 3′ UTR, as detected using a luciferase construct containing the potential 3′ UTR binding sites. Overall, this study established the first hypothalamic miRNA that directly targets the 3′ UTR of mouse *Npy*, emphasizing the involvement of miRNAs in the NPY system and providing an alternative target for control of NPY levels.

## 1. Introduction

Obesity, characterized by an excessive accumulation of body fat, is correlated with lower life expectancy and the development of comorbidities, including type II diabetes, cancer, and cardiovascular diseases [1]. The steady increase in the global prevalence of obesity demands efforts to address the unmet medical needs of affected individuals by uncovering novel therapeutic targets in obesity pathogenesis [2]. 

Within the brain, the hypothalamus serves as a central regulator of energy balance. The hypothalamic arcuate nucleus (ARC) contains orexigenic neuropeptide Y and agouti-related peptide (NPY/AgRP) neurons and anorexigenic proopiomelanocortin (POMC) neurons that sense and respond to hormonal and nutritional signals from the periphery to modulate feeding behavior and energy expenditure [3]. In the context of energy homeostasis, the primary function of hypothalamic NPY is to increase feeding behavior and decrease energy expenditure [4]. As a potent orexigen, NPY can lead to hyperphagia and weight gain when in excess, while NPY inhibition reduces body weight in animal models [5,6,7]. NPY has been previously shown to be dysregulated with obesogenic stimuli, including the ubiquitous endocrine disruptor bisphenol A (BPA) and high-fat diet (HFD) [8,9,10,11]. Taken together, targeting NPY could be a plausible intervention for human obesity. As such, understanding mechanisms contributing to the overall regulation of NPY, including any post-transcriptional mechanisms, is of particular interest to uncover candidates with greater therapeutic potential. 

One possible method to modulate NPY levels would be to employ a class of non-coding RNA molecules, microRNAs (miRNAs). miRNAs contain approximately 22 nucleotides and bind to the 3′ untranslated region (UTR) of target mRNAs to suppress gene expression through mRNA degradation and/or translational inhibition [12,13]. An increasing number of clinical studies have described a difference in circulating and tissue-specific miRNA profiles between obese and non-obese individuals, revealing a plausible involvement of miRNAs in the pathogenesis of obesity [14,15,16,17,18]. Moreover, miRNAs in the hypothalamus are indispensable to the central control of energy homeostasis [19,20]. Deletion of Dicer, a key miRNA-processing enzyme, in the ARC of mice, results in miRNA deficiency, accompanied by altered neuropeptide expression and hyperphagic obesity [20]. Hypothalamic expression of Dicer changes in response to nutrient availability as fasted mice exhibit elevated levels of Dicer mRNA in the hypothalamus, while obese mice show lower Dicer expression under the same fasting conditions [19]. In mice and rats, differential hypothalamic miRNA profiles between lean and obese animals are reported in several studies [21,22,23]. Altogether, this suggests that miRNA expression and the resulting miRNA-mediated regulation of genes in the hypothalamus may be altered in the obese state, further highlighting the physiological relevance of hypothalamic miRNA in energy homeostasis. Specifically, there is little information on miRNAs that may directly bind to and regulate *Npy* expression.

Previous work in our laboratory has identified three miRNAs that indirectly alter *Npy* expression [24,25]. Using the obesogenic compound BPA and the saturated fatty acid palmitate, which are potent inducers of *Npy*, McIlwraith et al. sought to identify miRNAs that were concurrently altered with *Npy* expression [24,25]. Of interest, miR-708-5p, one of the miRNAs increased by BPA, modulates *Npy* expression as miR-708-5p overexpression increases *Npy* mRNA [25]. Furthermore, palmitate increased miR-2137 and decreased miR-503-5p levels in hypothalamic cell models, and the overexpression of miR-2137 and miR-503-5p upregulated and downregulated *Npy* mRNA levels, respectively [24]. Since miRNAs primarily act as repressors of gene expression, miR-708-5p and miR-2137 likely increase *Npy* through an indirect mechanism that does not involve direct binding to the *Npy* 3′ UTR. This is corroborated by the fact that a predicted binding site for miR-708-5p, miR-2137, and miR-503-5p does not exist in the *Npy* 3′ UTR. The exact mechanisms underlying these effects are not known. Moreover, to the best of our knowledge, hypothalamic miRNA(s) with direct binding ability to the *Npy* 3′ UTR have not been described to date. 

In this study, we used a miRNA target prediction algorithm to generate a list of candidates that could possibly bind the mouse *Npy* 3′ UTR. We also found that a subset of these miRNAs, including miR-143-3p, was altered by BPA in defined hypothalamic neuronal models. We then investigated the effects of overexpressing selected miRNA candidates on *Npy* expression in clonal hypothalamic cell lines and reported downregulation of *Npy* levels in mHypoE-41 cells transfected with miR-143-3p, miR-140-5p, or let-7b-3p mimic. Subsequent luciferase assays confirmed that miR-143-3p, but not miR-140-5p or let-7b-3p, bound to the *Npy* 3′ UTR. Hence, we are the first that we know to describe a hypothalamic miRNA that binds to the *Npy* 3′ UTR and represses *Npy* mRNA. 

## 2. Materials and Methods

### 2.1. Cell Culture and Conditions

Clonal hypothalamic female-derived mHypoE-41 and mHypoA-59, as well as male-derived mHypoE-46, mHypoE-42, mHypoE-44, and mHypoA-2/12 neuronal cell lines were previously generated by immortalizing neurons derived from embryonic (E) or adult (A) mice, express NPY, and have been fully characterized [26,27]. The mHypoE-41, mHypoA-59, mHypoE-42, mHypoE-44, and mHypoA-2/12 cells were cultured in Dulbecco’s Modified Eagle Medium (DMEM; MilliporeSigma, Oakville, ON, Canada) with 4500 mg/L glucose, supplemented with 2% fetal bovine serum (FBS; Gibco, Burlington, ON, Canada) and 1% penicillin-streptomycin (PS; Gibco). The mHypoE-46 cells were cultured in DMEM (MilliporeSigma, Oakville, ON, Canada) with 1000 mg/L glucose, supplemented with 5% FBS (Gibco) and 1% PS (Gibco). All cells were incubated at 37 °C with 5% CO_2_. At the time of 24 h prior to BPA treatment and miRNA mimic transfection, cells were split into 60 mm plates and were grown to 70–80% confluency. Growth media were replaced with treatment or transfection media on the day of the experiment. 

Primary culture protocols were conducted in compliance with the regulations of the Canadian Council on Animal Care and approved by the University of Toronto Animal Care Committee. Cells from the hypothalamii of 8-week-old male CD-1 mice were collected for primary culture and were triturated into 6-well plates. The cells were cultured in neurobasal A medium (Gibco), supplemented with 10% FBS, 5% horse serum (Gibco), 1% PS (Gibco), 1 × B27 supplement (Gibco), and 1 × GlutaMAX supplement (Gibco) for approximately 9 days. An amount of 10 ng/µL ciliary neurotrophic factor (CNTF; R&D Biosystems, Oakville, ON, Canada) was applied to each well daily to induce proliferation. Growth media was replaced with treatment media (as follows below) on the day of the experiment. 

### 2.2. BPA Treatment 

Bisphenol A (BPA; MilliporeSigma) was prepared by dissolving anhydrous ethyl alcohol (EtOH) to a concentration of 200 mM, which was further diluted 1:1 in sterile water to achieve a stock concentration of 100 mM. Vehicle (50% EtOH + 50% H_2_O) or 100 mM BPA solution was then diluted 1:1000 in 4500 mg/L glucose-containing phenol red–free DMEM (Hyclone Laboratories Inc., Whitby, ON, Canada), supplemented with 1% charcoal-dextran stripped FBS (CSFBS; Gemini Bio Products, Burlington, ON, Canada) and 1% PS (Gibco), obtaining a final treatment concentration of 0.05% EtOH or 100 μM BPA. Growth media were removed, and cells were washed with phosphate-buffered saline (PBS). Treatment media containing either vehicle (0.05% EtOH) or 100 μM BPA were added to cells for 16 or 24 h. Similarly, primary cultures were treated with 0.05% EtOH or 100 μM BPA for 16 h. 

### 2.3. miRNA Mimic Transfection 

For 24 h miRNA mimic transfection, mHypoE-46 and mHypoE-41 cells were grown to 70–80% confluency in 60 mm tissue culture plates. mirVana miRNA mimic negative control #1 (ID: 4464061), miR-29b-1-5p mimic (ID: MC12431), miR-140-5p mimic (ID: MC10205), miR-143-3p mimic (ID: MC10883), let-7f-1-3p (ID: MC13032), and let-7b-3p (ID: MC12489) were purchased from Thermo Fisher Scientific (Burlington, ON, Canada). For transient transfection, 25 nM mimic or negative control solutions were complexed with Dharmafect 3 Transfection Reagent (Dharmacon, Cedarlane, Burlington, ON, Canada) for 20 min at room temperature in serum- and antibiotic-free plain DMEM with 4500 mg/L glucose (Millipore Sigma). The complexed transfection mixture was then diluted 1:10 with antibiotic-free DMEM with 4500 mg/L glucose (Millipore Sigma) supplemented with 2% FBS. Cells were washed with PBS, and the transfection media was added to the cells for 24 h. 

### 2.4. RNA Isolation, cDNA Synthesis, and Quantitative RT-PCR 

For hypothalamic cell line experiments, RNA isolation was performed with the Total RNA Purification Kit (Norgen Biotek Corp, Thorold, ON, Canada), which included an on-column DNAse procedure for genomic DNA removal. Primary culture RNA was isolated with the PureLink RNA isolation kit (Thermo Fisher Scientific) by following the manufacturer’s instructions, and genomic DNA was removed through the on-column PureLink DNase step (Thermo Fisher Scientific). RNA was quantified with the NanoDrop 2000 (Thermo Fisher Scientific). To study mRNA expression, 500–1000 ng RNA was used to synthesize complementary cDNA (cDNA) using the High-Capacity cDNA Reverse Transcription Kit (Thermo Fisher Scientific). For miRNA analysis, 100 ng of total RNA was used to synthesize miRNA cDNA using the dNTP from High-Capacity cDNA Reverse Transcription Kit (0.1 mM) (Thermo Fisher Scientific), adenosine triphosphate ATP (0.1 mM) (New England Biolabs Ltd., Whitby, ON, Canada), *Escherichia coli* (*E. coli*) Poly(A) Polymerase (100 U/mL) (New England Biolabs Ltd., Whitby, ON, Canada), and M-MLV Reverse Transcriptase (10 U/µL) (Thermo Fisher Scientific) or MultiScribe Reverse Transcriptase (10 U/µL) [28]. miRNA primers were designed with the help of miRPrimer, using consensus miRNA sequence from miRBase (accessed November 2022) [29].Quantitative reverse transcription polymerase chain reaction (RT-qPCR) was performed using PowerTrack SYBR Green Master Mix (Thermo Fisher Scientific) and gene (0.4 µM/reaction) or miRNA (2 µM/reaction) specific primers (Table 1) on the QuantStudio 5 (Thermo Fisher Scientific) with the following setup: 2 min at 95 °C, 40 cycles of 5 s at 95 °C and 30 s at 60 °C, and melt curve analysis (15 s at 95 °C, 1 min at 60 °C, 15 s at 95 °C). Data were analyzed with the ΔΔCT method and normalized to the reference gene, 60S ribosomal protein L7 (Rpl7), or reference miRNA, miR-221-3p [29,30]. We established miR-221-3p as an endogenous control because its levels are not changed by BPA [25]. 

### 2.5. In Silico Identification of miRNA Candidates

Bioinformatics prediction of miRNA targets was conducted using TargetScanMouse 8.0 to retrieve a list of miRNAs with *Npy* as a potential target [31,32]. miRNAs with predicted binding sites on the 3′ UTR of *Npy* mRNA were identified as candidate miRNAs. Based on Affymetrix GeneChip miRNA 4.0 array performed by our lab on the whole hypothalamus and immortalized hypothalamic cell lines (unpublished data), miRNAs that were significantly detected above background signal (DABG *p* < 0.05) in the whole hypothalamus or immortalized hypothalamic neurons were defined as confidently expressed. Moreover, miRNA candidates were further sorted by annotation confidence according to miRbase to avoid misannotation of siRNAs or fragments of other RNAs as miRNAs [33,34]. The following requirements were established by miRbase to determine high-confidence miRNAs: more than 10 reads map to the two possible mature miRNAs derived from hairpin precursor with no mismatch; at least half of the reads mapping to each arm of hairpin precursor have an identical 5′ end; a minimum of 60% of the bases in the mature sequences are paired in the putative hairpin structure; the most abundant reads at each arm of the hairpin precursor are paired in the mature miRNA duplex with 0–4 bp overhang at the 3′ end; the folding free energy of the putative hairpin structure is less than −0.2 kcal/mol/nt. 

### 2.6. Npy 3′ Untranslated Region Construct Synthesis 

The predicted 180 bp of the 3′ UTR of the *Npy* gene, as determined by TargetScanMouse 8.0, was amplified from mouse genomic DNA (Genomic DNA Purification Kit, Thermo Fisher Scientific) with primers designed to incorporate restriction enzyme sites complementary to the multiple cloning site of the pmirGLO vector (Promega Corp, Madison, WI, USA). Primers: Npy 3′ UTR + SacI site (bold letters) forward 5′ TTGTCTGCAT**GAGCTC**TGGGAAATGAAACTTGTTCT 3′ and Npy 3′ UTR + SalI site (bold letters) reverse 5′ CGGTCTGACCC**GTCGAC**TTTTGAATGCATGGTACTTT 3′. After restriction digest of both the vector and 3′ UTR with SacI and SalI, the two were ligated with Quick Ligation™ Kit (New England BioLabs Ltd.) and cloned into DH5α-competent cells. Ampicillin-resistant bacterial colonies were selected and grown to isolate DNA. The presence of the insert in the correct orientation was confirmed by running restriction-digested products on a 1% agarose gel (Thermo Fisher Scientific) and Sanger sequencing. Restriction enzymes and buffers were purchased from New England BioLabs Ltd. Vector DNA containing the *Npy* 3′ UTR insert was sent for sequencing at the Centre for Applied Genomics.

### 2.7. Construct and miRNA Mimic Co-Transfection for Dual Luciferase Reporter Assay 

At the time of 24 h prior to reporter plasmid and miRNA mimic co-transfection, mHypoE-41 cells were split and cultured in 6-well plates to 70–80% confluency. mHypoE-41 cells were transfected with 1 μg of pmiRGLO-Empty or *Npy* 3′ UTR and 25 nM of indicated miRNA mimic or negative control using 6 μL of Turbofect (Thermo Fisher Scientific) per well for 24 h. Two technical replicates were conducted for each biological replicate. Cells were washed once with 1 × PBS and were then lysed in 500 μL passive lysis buffer (Promega Corp) per well. The cell lysates were used to determine luciferase activity using a dual luciferase reporter assay kit (Promega Corp) according to the manufacturer’s instructions. 

### 2.8. Statistical Analyses

GraphPad Prism 7.0 (GraphPad Prism Software Inc., Boston, MA, USA) was utilized to conduct data analysis and determine statistical significance. A student’s *t*-test or two-way ANOVA, followed by Tukey’s multiple comparison test, was performed as indicated in the figure legends. Each experiment was conducted with at least *n* = 3 biological replicates, as in the figure legends. *p* ≤ 0.05 was used as the threshold for statistical significance. Data are represented as mean ± standard error of the mean (SEM). Statistical significance between vehicle and treatment was presented as * *p* ≤ 0.05, ** *p* < 0.01, *** *p* < 0.001, and **** *p* < 0.0001. 

## 3. Results

### 3.1. Identification of Five Putative Npy-Targeting miRNAs through In Silico and In Vitro Analyses

**Figure 1:**
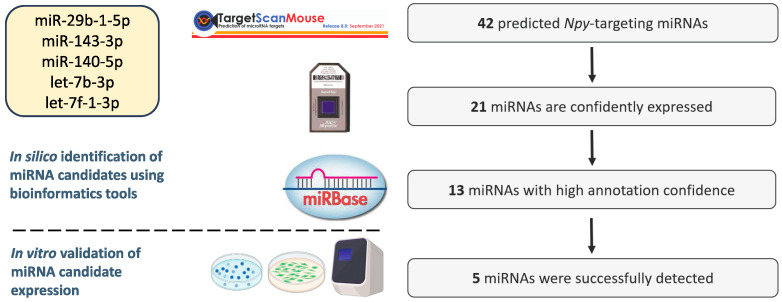
Flow diagram illustrating the selection criteria for candidate miRNAs. TargetScan was used to generate a list of miRNAs with putative binding sites on the 3′ UTR of *Npy*. Using hypothalamic miRNA array data, miRNAs with detection above background (DABG) values (*p* < 0.05) in the whole hypothalamus or immortalized hypothalamic neurons were defined as confidently expressed. Annotation confidence determined by miRBase was used to further eliminate misannotation of siRNAs or fragments of other RNAs as miRNAs. The expression of miRNA candidates was validated in hypothalamic cell lines and primary culture. Eventually, 5 putative *Npy*-targeting miRNAs were detected in the hypothalamic cell models. BioRender.com (accessed on 17 August 2023) was used to create the figure.

**Figure 2:**
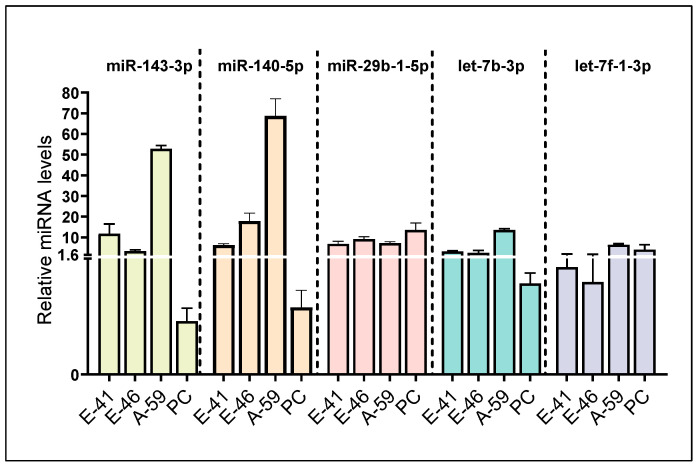
Comparison of the miRNA gene expression in the hypothalamic neuronal models. Basal expression of miR-143-3p, miR-140-5p, miR-29b-1-5p, let-7b-3p, and let-7f-1-3p levels were assessed in mHypoE-41 (E-41), mHypoE-46 (E-46), mHypoA-59 (A-59) neurons and primary cultures of male hypothalamic neurons (PC) (*n* = 3–4). The expression of candidate miRNAs is normalized to miR-221-3p. Data are represented as mean ± SEM.

To the best of our knowledge, a hypothalamic miRNA that can directly bind to the 3′ UTR of the *Npy* gene has not been reported in the literature. More than 60% of human protein-coding genes are regulated by miRNAs, and accumulating studies have demonstrated that multiple miRNAs can regulate a single gene [35,36,37,38]. Hence, we aimed to closely examine whether *Npy*, an orexigenic neuropeptide, is modulated by miRNAs. To this end, we utilized bioinformatics tools to initially identify miRNA candidates, which were then subject to in vitro validation. In the present study, a total of 42 miRNAs that could potentially bind to the *Npy* 3′ UTR were identified from the miRNA target prediction database TargetScan (Figure 1). This list was further refined to obtain high-confidence predictions based on microarray profiling data and miRBase-derived annotation confidence (Table 2, Figure 1) [33,34]. By doing so, this reduces the chance of selecting candidates that are either misannotated as miRNAs or not expressed in the hypothalamic cell lines. To ensure miRNA candidates were expressed in the hypothalamus, clonal hypothalamic immortalized *Npy*-expressing mHypoE-41, mHypoE-46, and mHypoA-59 cells, as well as hypothalamic neuronal primary cultures, representing a mixed population of non-immortalized hypothalamic neurons, were utilized to validate the presence of miRNA candidates in models of hypothalamic neurons. Ultimately, the expression of the following 5 miRNAs, miR-143-3p, miR-140-5p, miR-29b-1-5p, let-7b-3p, and let-7f-1-3p, were successfully detected through RT-qPCR (Figure 2). 

In primary cultures of hypothalamic neurons, consisting of an undefined heterogeneous mixture of neuronal subtypes, the levels of miR-29b-1-5p and let-7f-1-3p were modestly higher than other miRNA candidates (Figure 2). The basal expression of miR-29b-1-5p was largely comparable between primary culture and immortalized hypothalamic cells (Figure 2). Moreover, *Npy*-expressing cell lines generally expressed higher levels of miR-140-5p, miR-143-3p, and let-7b-3p compared to the primary culture, implying that certain miRNAs may be enriched in NPY neurons compared to the heterogeneous cultures (Figure 2). Interestingly, miRNA expression profiles varied between *Npy*-expressing cell lines as the basal levels of individual miRNAs were different, implying that each miRNA within a cell line may exert distinct regulation of target genes. 

### 3.2. BPA Alters mRNA Levels of miRNA Biogenesis Components in the Hypothalamic-Derived Neuronal Cell Lines

Regulation of components of the miRNA machinery by estradiol was reported previously [39]. Due to the estrogenic properties of BPA, we sought to elucidate the impact of BPA on components belonging to the miRNA biosynthesis pathway in the hypothalamic cell lines mHypoE-46, mHypoE-41, and mHypoA-59 that were treated with 100 µM BPA for 24 h. The expression of miRNA processing enzyme components (*Drosha*, *Dgcr8*, *Dicer*) and a critical component of the RNA-induced silencing complex (RISC), *Ago2*, were assessed. 

**Figure 3:**
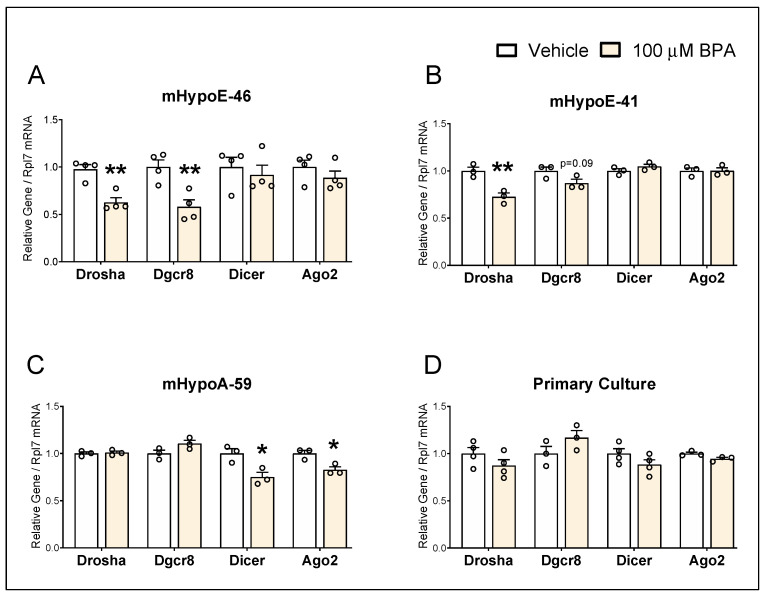
BPA exposure dysregulates miRNA machinery genes in the hypothalamic neuronal models. (**A**) mHypoE-46 cells (*n* = 4), (**B**) mHypoE-41 cells (*n* = 3), and (**C**) mHypoA-59 cells (*n* = 3) were treated with 100 µM BPA or 0.05% EtOH for 24 h. (**D**) Primary cultures of hypothalamic neurons (*n* = 3–4) were treated with 100 µM BPA or 0.05% EtOH for 16 h. The mRNA levels of *Drosha*, *Dgcr8*, *Dicer,* and *Ago2* were assessed. Data are represented as mean ± SEM. Statistical significance was determined by the student’s *t*-test and is represented as * *p* ≤ 0.05, and ** *p* < 0.01.

*Drosha*, *Dgcr8*, *Dicer,* and *Ago2* were expressed in all three cell lines (Figure 3), providing evidence that these hypothalamic cell lines possess essential components of miRNA machinery required for endogenous miRNA biogenesis. In the mHypoE-46 cells, the mRNA expression of *Drosha* and *Dgcr8* was decreased by BPA, whereas *Dicer* and *Ago2* were not significantly altered (Figure 3A). Only *Drosha* was significantly downregulated by BPA in the mHypoE-41 cells, while no significant changes were observed in other miRNA machinery components (Figure 3B). As opposed to the mHypoE-46 and mHypoE-41 cells, neither *Drosha* nor *Dgcr8* was changed by BPA in the mHypoA-59 cells (Figure 3C). In contrast, BPA-mediated downregulation of *Dicer* and *Ago2* was observed in the mHypoA-59 cells (Figure 3C), and these effects were absent in the mHypoE-46 and mHypoE-41 cells (Figure 3A,B). However, none of the miRNA biogenesis components were altered by BPA in the primary cultures of hypothalamic neurons at the mRNA level (Figure 3D). These results illustrate that not all miRNA machinery components were equally affected by BPA and the pleiotropic effects of BPA on *Npy*-expressing subpopulations were mimicked in the context of miRNA processing components. 

### 3.3. BPA Induces Differential miRNA Changes in Hypothalamic-Derived Neuronal Cell Lines (mHypoE-41, mHypoE-46, mHypoE-44, mHypoE-42, mHypoA-59, and mHypoA-2/12) and in Neuronal Primary Culture 

Our lab has previously shown that BPA treatment alters *Npy* mRNA levels differentially in unique clonal hypothalamic neuronal cell models [9]. The heterogeneous nature of the NPY neurons, due to their multifaceted functional nature, is discussed therein and has been documented previously in vivo and in vitro [9,40,41,42]. It has also been reported that BPA exposure in humans is associated with changes in circulating miRNA expression [43], and mice exposed to BPA in utero display altered miRNA expression profiles within the hypothalamus [44,45]. The potential of BPA to affect hypothalamic miRNAs is further corroborated by disrupted miRNA signatures in our hypothalamic cell lines upon BPA treatment [25]. 

**Figure 4:**
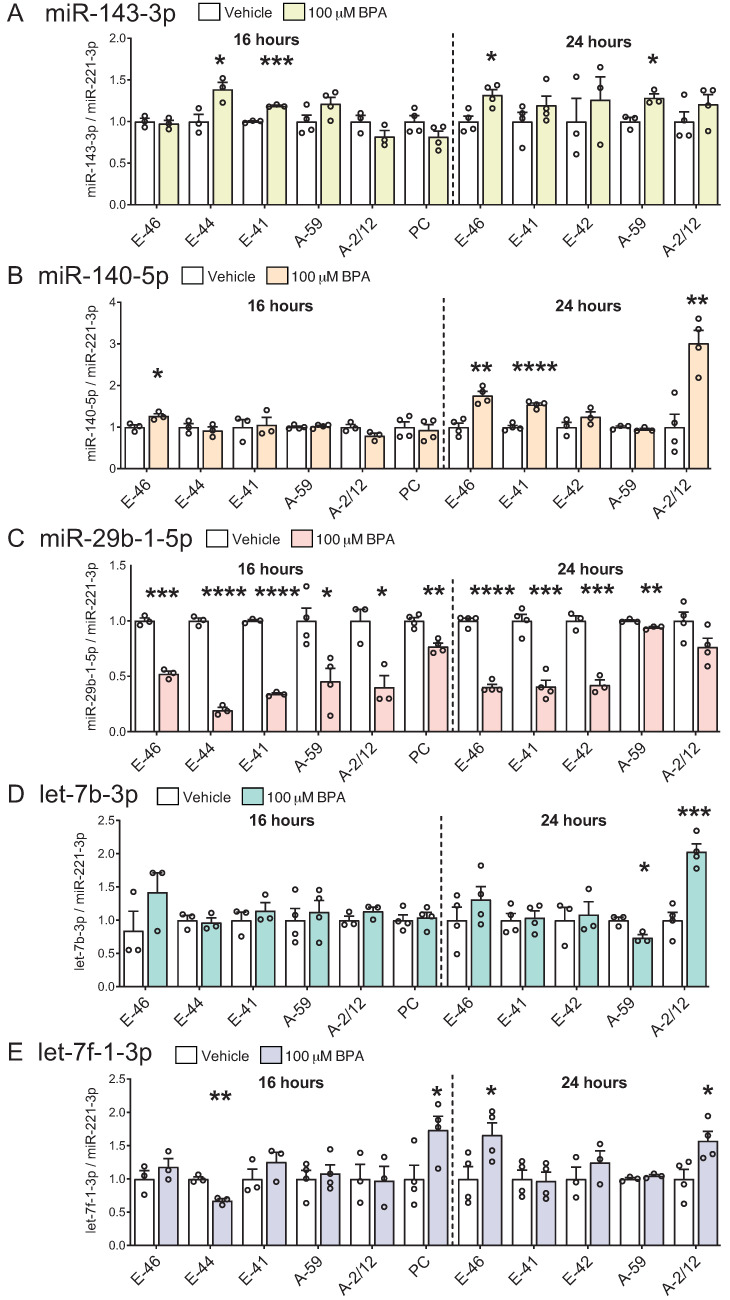
BPA alters putative *Npy*-targeting miRNAs in hypothalamic neuronal models and primary culture. mHypoE-46 (E-46), mHypoE-44 (E-44), mHypoE-41 (E-41), mHypoE-42 (E-42), mHypoA-59 (A-59), mHypoA-2/12 (A-2/12) neurons were treated with 100 µM BPA (colored bars) or 0.05% EtOH (vehicle, white bars) for 16 or 24 h. Primary cultures of male hypothalamic neurons (PC) were treated for 16 h with 100 µM BPA or 0.05% EtOH (vehicle). Changes in (**A**) miR-143-3p, (**B**) miR-140-5p, (**C**) miR-29b-1-5p, (**D**) let-7b-3p, and (**E**) let-7f-1-3p levels were assessed in hypothalamic neuronal models following BPA exposure (*n* = 3–4). Data are represented as mean ± SEM. Statistical significance was determined by the student’s *t*-test and is represented as * *p* ≤ 0.05, ** *p* < 0.01, *** *p* increase < 0.001, and **** *p* < 0.0001.

To investigate the effects of BPA on selected miRNA candidates, the *Npy*-expressing mHypoE-41, mHypoE-46, mHypoE-44, mHypoE-42, mHypoA-59, and mHypoA-2/12 cells were treated with 100 µM BPA for 16 or 24 h (Figure 4A–E). Many of the miRNAs were differentially altered by BPA in the individual neuronal subpopulations, similar to that seen with the miRNA biosynthesis components. miR-143-3p was upregulated by BPA in the mHypoE-44, mHypoE-46, mHypoE-41 and mHypoA-59 neurons, but there were no significant changes in the mHypoA-2/12 and mHypoE-42 neurons (Figure 4A). miR-140-5p was exclusively upregulated in the mHypoE-46, mHypoE-41 and mHypoA-2/12 cells (Figure 4B). On the other hand, miR-29b-1-5p levels were consistently downregulated by BPA in all hypothalamic cell lines at 16 h, and miR-29b-1-5p was repressed up to ~80% in the mHypoE-44 cells (Figure 4C). At 24 h, the magnitude of repression was also highly significant in the mHypoE-46, mHypoE-41, and mHypoE-42 cells as miR-29b-1-5p was decreased by around 60% in these cell lines (Figure 4C). While let-7b-3p was downregulated in the mHypoA-59 cells, let-7b-3p was upregulated in the mHypoA-2/12 cells (Figure 4D). Similarly, let-7f-1-3p was downregulated in the mHypoE-44 cells but upregulated in the mHypoE-46 and mHypoA-2/12 (Figure 4E). 

Next, to establish whether these changes were present in a non-immortalized heterogeneous mixed culture of hypothalamic neurons, we treated the hypothalamic primary culture with BPA for 16 h. The BPA-mediated downregulation of miR-29b-1-5p in the hypothalamic cell lines was preserved in the primary culture model, with an approximately 23% decrease in response to BPA (Figure 4C). The levels of let-7f-1-3p were elevated by 73% in the BPA-treated primary culture, which was comparable to a 66% increase in let-7f-1-3p in the mHypoE-46 cells (Figure 4E). Overall, these findings illustrate differential modulation of hypothalamic miRNAs by BPA in unique neuronal subpopulations.

### 3.4. Overexpression of miR-143-3p, miR-140-5p and let-7b-3p Downregulate Npy mRNA in the Hypothalamic Npy-Expressing mHypoE-41 Cell Model

**Figure 5:**
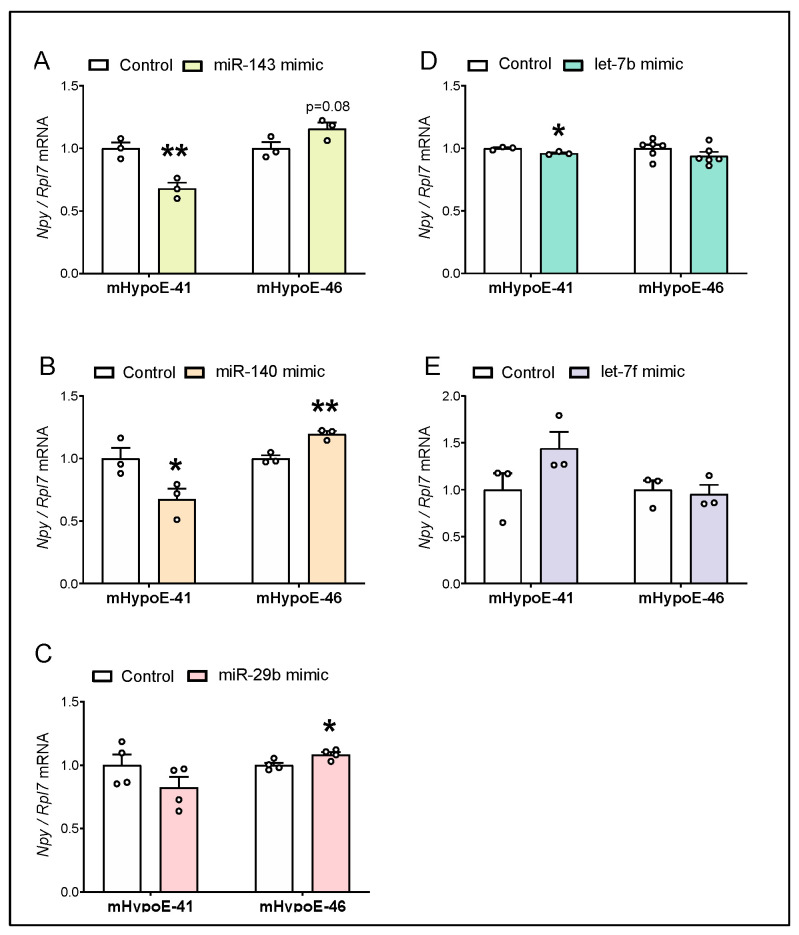
Altered *Npy* mRNA levels by overexpression of putative *Npy*-targeting miRNAs in hypothalamic cell lines. mHypoE-41 and mHypoE-46 cells were treated with negative control or (**A**) miR-143-3p, (**B**) miR-140-5p, (**C**) miR-29b-1-5p, (**D**) let-7b-3p, (**E**) let-7f-1-3p mimic for 24 h (*n* = 3–6). Data are represented as mean ± SEM. Statistical significance was determined by student *T*-test and is represented as * *p* ≤ 0.05, and ** *p* < 0.01.

Since miR-143-3p, miR-140-5p, miR-29b-1-5p, let-7b-3p, and let-7f-1-3p were detected in models of hypothalamic neurons, we next aimed to investigate the role of miRNA candidates in regulating *Npy* expression. To examine the impact of miRNA candidates on *Npy* levels, two hypothalamic cell models were transfected with 25 nM miRNA mimics for 24 h to overexpress individual miRNA candidates (Figure 5A–E). As shown in Figure 5A and 5B, the levels of *Npy* in mHypoE-41 cells transfected with miR-143-3p and miR-140-5p mimics were repressed by 32% and 33%, respectively. *Npy* expression was modestly downregulated by 4% in response to let-7b-3p overexpression in mHypoE-41 cells (Figure 5D). No significant change in *Npy* was observed in the mHypoE-41 cells with miR-29b-1-5p and let-7f-1-3p overexpression, compared to the cells transfected with negative control (Figure 5C,E). 

With the goal of understanding the overall involvement of miRNAs in the regulation of *Npy*, we also transfected the mHypoE-46 cells with miRNA mimics. Given the diverse functions of hypothalamic NPY neurons, we hypothesized that miRNA-mediated regulation of *Npy* may be different between *Npy*-expressing neuronal subpopulations. In the case of mHypoE-46 cells, none of the miRNA mimics reduced *Npy* expression, suggesting a mechanistic differences in the regulation of *Npy* by miRNAs between mHypoE-41 and mHypoE-46 neurons (Figure 5). In fact, *Npy* expression was increased by miR-140-3p and miR-29b-1-5p overexpression in the mHypoE-46 cells (Figure 5B,C), and unchanged by miR-143-3p, let-7b-3p, and let-7f-1-3p (Figure 5A,D,E). Taken together, these data indicate that *Npy* reduction by miRNA mimics was specific to the mHypoE-41 cells.

### 3.5. miR-143-3p Targets the Npy 3′ UTR in mHypoE-41 Neurons

**Figure 6:**
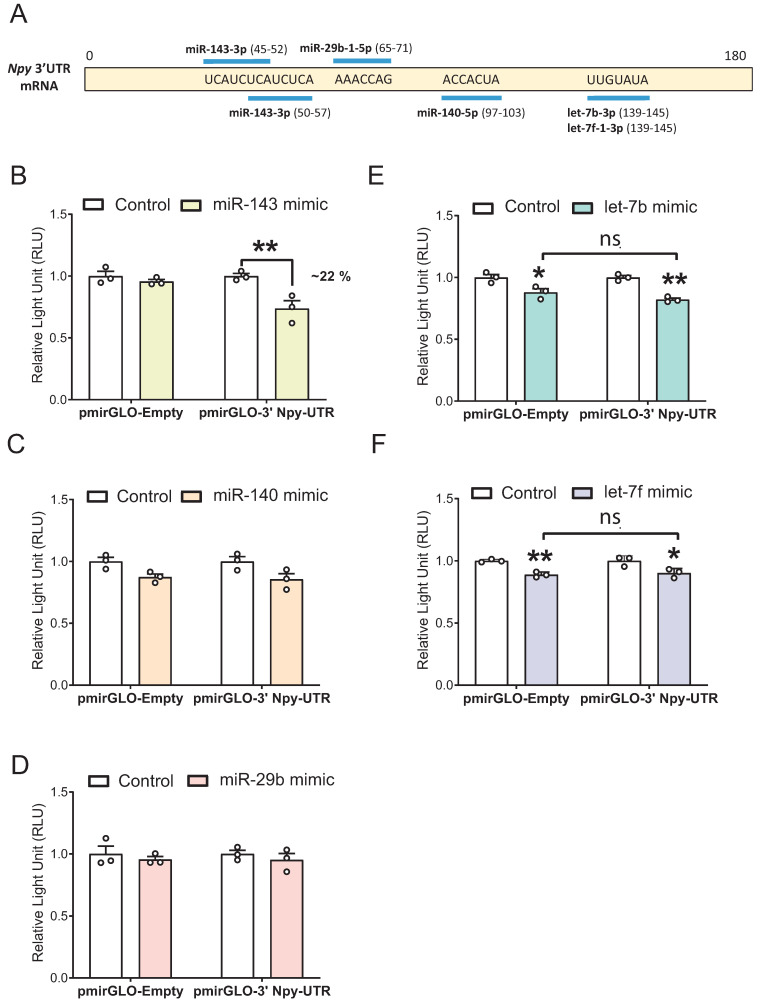
miR-143-3p targets *Npy* 3′ UTR in mHypoE-41 neurons. (**A**) Binding sites of miRNA candidates on *Npy* 3′ UTR. Co-transfection of pmirGLO vector (1 µg) containing 180 bp 3′ *Npy*-UTR sequence with 25 nM of (**B**) miR-143-3p mimic (**C**) miR-140-5p mimic, (**D**) miR-29b-1-5p mimic, (**E**) let-7b-3p mimic, and (**F**) let-7f-1-3p mimic for 24 h (*n* = 3 independent experiments/group, each run in duplicate). Data are represented as mean ± SEM. Statistical significance was determined by two-way ANOVA, followed by the Tukey multiple comparison test; Data are represented as * *p* ≤ 0.05, and ** *p* < 0.01.

Our next question was whether miR-143-3p, miR-140-5p, miR-29b-1-5p, let-7b-3p, and let-7f-1-3p directly target the 3′ UTR of *Npy* since targeting can occur without altering mRNA expression. According to in silico target prediction databases, two putative binding sites were identified for miR-143-3p, while miR-140-5p, miR-29b-1-5p, let-7b-3p, and let-7f-1-3p putatively bind to one site in the mouse *Npy* 3′ UTR, as illustrated (Figure 6A). Both let-7b-3p and let-7f-1-3p belong to the let-7 family and are predicted to bind to the same position on the *Npy* 3′ UTR. To verify if our miRNA candidates could bind to the *Npy* 3′ UTR, dual-luciferase reporter assays were performed to assess the effects of miRNA mimics on the reporter activity of the pmirGLO luciferase vector containing the mouse 3′ *Npy*-UTR. To this end, cells were transiently transfected with the pmirGLO vector containing the *Npy* 3′ UTR (pmirGLO-3′ Npy-UTR) along with individual miRNA mimic or negative control for 24 h, and changes in luciferase activity were measured. 

A 22% decrease in luciferase signal from the pmirGLO-3′ Npy-UTR construct was observed in cells transfected with the miR-143-3p mimic compared with negative control after 24 h (Figure 6B). Transfection of miR-140-5p or the miR-29b-1-5p mimic did not result in significant changes in luciferase activity of *Npy* 3′ UTR reporter plasmid compared to negative control (Figure 6C,D). Finally, although the luciferase output of pmirGLO-3′ Npy-UTR was decreased by the let-7b-3p and let-7f-1-3p mimics, it also reduced luciferase activity of pmirGLO-Empty, suggesting that let-7b-3p and let-7f-1-3p may bind to or affect other factors regulating the empty plasmid itself (Figure 6E,F). Therefore, it remains inconclusive whether *Npy* is a direct binding target of let-7b-3p and let-7f-1-3p. Altogether, these results conclusively demonstrated that miR-143-3p directly targets *Npy* 3′ UTR to regulate the expression of *Npy* in immortalized hypothalamic mHypoE-41 neurons. 

### 3.6. miRNA Candidates Alter Estrogen Receptor Genes and Metabolism-Related Genes 

All five miRNA candidates investigated herein have been implicated in energy homeostasis, either peripherally or centrally. In addition to *Npy*, these miRNA candidates may also modulate other genes to influence the regulation of energy balance by hypothalamic neurons. Furthermore, differential regulation of *Npy* has been previously linked to estrogen receptor (ER) levels, as such miRNA-mediated changes in the ER levels may play a role in differential *Npy* regulation observed. Hence, the potential relevance of individual miRNA candidates on *Esr1* and *Esr2* expression was assessed. 

**Figure 7:**
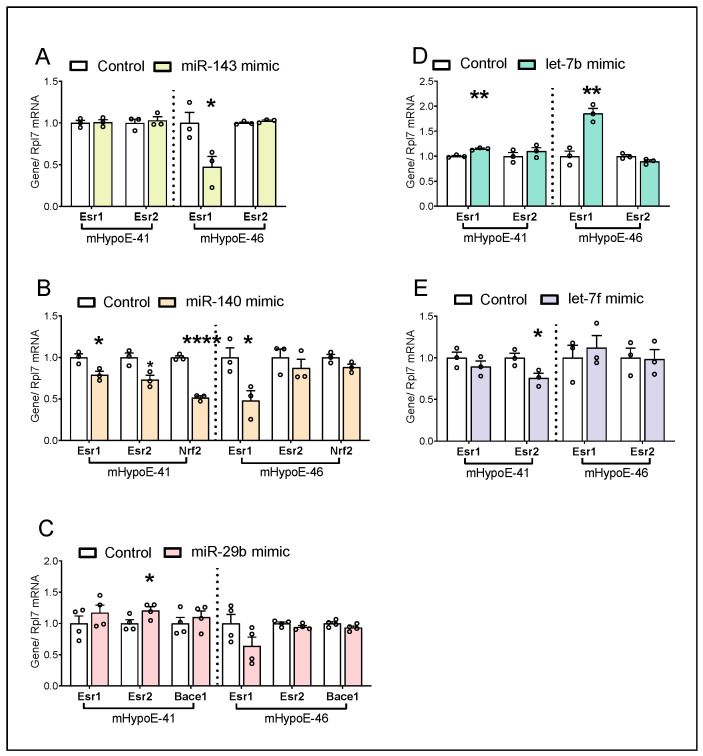
miRNA candidates regulate estrogen receptors and genes related to energy homeostasis. mHypoE-41 and mHypoE-46 cells were treated with negative control or 25 nM of (**A**) miR-143-3p, (**B**) miR-140-5p, (**C**) miR-29b-1-5p, (**D**) let-7b-3p, (**E**) let-7f-1-3p mimic for 24 h (*n* = 3–4). Estrogen receptors (*Esr1* and *Esr2*), a metabolism-related gene (*Bace1*)*,* and an oxidative stress-related gene (*Nrf2*) were evaluated. Data are represented as mean ± SEM. Statistical significance was determined by the student’s *t*-test and is represented as * *p* ≤ 0.05, ** *p* < 0.01, and **** *p* < 0.0001.

Although *Esr1* and *Esr2* were not predicted targets of miR-143-3p and miR-140-5p, given the dramatic differences seen in *Npy* regulation by these mimics in the two cell lines, we sought to determine the effects of these mimics on the ERs. *Esr1* downregulation by the miR-143-3p mimic was observed in the mHypoE-46 cells only, while *Esr1* downregulation by the miR-140-5p mimic was seen in both cell lines (Figure 7A,B). *Esr2* was not affected by the miR-143-3p mimic in both cell lines (Figure 7A), while *Esr2* was downregulated by the miR-140-5p mimic in the mHypoE-41 cells only (Figure 7B). *Esr1* and/or *Esr2* were predicted targets of miR-29b-1-5p, let-7b-3p, and let-7f-1-3p. In terms of miR-29b-1-5p mimic, *Esr2* was upregulated in the mHypoE-41 cells but not the mHypoE-46 cells, while *Esr1* was not affected by the miR-29b-1-5p mimic in either cell line (Figure 7C). Unexpectedly, increased *Esr1* expression by the let-7b-3p mimic was observed in both mHypoE-41 and mHypoE-46 cells, with greater induction of *Esr1* in the latter (Figure 7D). While *Esr2* was not affected by the let-7b-3p mimic, *Esr2* was decreased by the let-7f-1-3p mimic in the mHypoE-41 but not the mHypoE-46 cells (Figure 7D,E). Intriguingly, increased *Npy* expression correlates with a higher *Esr2*/*Esr1* ratio in the mHypoE-46 cells (Table 3). These results suggest that miRNA mimic-mediated changes to the ERb:ERa ratio may contribute to the reversal of *Npy* expression between mHypoE-41 and mHypoE-46 cells.

Additional miRNA targets that are related to metabolism were further explored. We measured the expression of a validated target of miR-140-5p, *Nrf2*, a transcription factor regulating antioxidant stress response [46,47]. Decreased *Nrf2* by the miR-140-5p overexpression was seen in the mHypoE-41 cells but not in the mHypoE-46 cells (Figure 7B). We also examined *Bace1* that is a predicted target of miR-29b-1-5p. Previous studies have shown that *Bace1* deletion or inhibition protects mice from diet-induced obesity [48,49]. *Bace1* was not altered by miR-29b-1-5p mimic in the mHypoE-41 and mHypoE-46 cells (Figure 7C).

## 4. Discussion

NPY neurons play an essential role in the central regulation of appetite and body weight. Multiple studies have demonstrated that abnormal alteration to NPY levels in the hypothalamus can be detrimental to whole-body energy homeostasis, highlighting the importance of maintaining appropriate NPY signals at the brain level [5,6,25,50]. However, the complete mechanism underlying the regulation of hypothalamic NPY has not been fully delineated, and this study aims to understand the complex regulatory layers of the NPY system through the lens of miRNAs. In the current study, we discovered a previously unknown *Npy*-targeting miRNA in the *Npy*-expressing hypothalamic cell lines, specifically, miR-143-3p interacts with the murine *Npy* 3′ UTR to downregulate *Npy* mRNA expression. Moreover, we found that hypothalamic miRNAs were altered by BPA across *Npy*-expressing cell lines and primary cultures, indicating that exposure to endocrine-disrupting chemicals can dysregulate miRNA expression and its downstream regulation of hypothalamic processes. 

Hypothalamic miRNAs can regulate feeding neuropeptides through both direct and indirect mechanisms, supported by accumulating evidence from our research and other groups. Direct regulation can occur when miRNA targets neuropeptide mRNA to modulate its stability or translation efficiency, resulting in mRNA decay or translational inhibition. For example, we described that miR-143-3p is a direct regulator of *Npy* as miR-143-3p targets the mouse *Npy* 3′ UTR. Derghal et al. also reported that appetite-suppressing *Pomc* mRNA is a direct target of miR-383, miR-384-3p, and miR-488 [51]. Another study also showed that in vivo knockdown of miR-383 or miR-384 restores ethanol-induced dysregulation of POMC at the mRNA and protein level [52]. On the other hand, indirect regulation involves miRNA targeting components of signaling pathways or transcription factors that control gene expression. We have previously shown that miR-708-5p, miR-2137, and miR-503-5p indirectly regulate *Npy* as transient overexpression of these miRNA altered *Npy* levels despite not being predicted to target the mouse *Npy* 3′ UTR. In the current study, overexpression of miR-140-5p, miR-29b-1-5p, and let-7b-3p altered *Npy* mRNA, but a direct binding of aforementioned miRNAs to the mouse *Npy* 3′ UTR is not observed in this study. This suggests that *Npy* may be an indirect target of miR-140-5p, miR-29b-1-5p, and let-7b-3p such that these miRNAs could act on other genes that would influence the expression of *Npy*. Regardless of the mode of miRNA-mediated regulation of target genes, changes to the hypothalamic milieu could alter miRNA-feeding neuropeptide interaction as hypothalamic miRNAs are responsive to hormones, chemicals, and nutrients [24,25,51,52]. 

Fasting decreases circulating miR-143-3p levels in humans [53]. Furthermore, fasting increases the hypothalamic expression of *Npy* mRNA in vivo [53]. As such, a decrease in miR-143-3p levels may enable the expression of *Npy* by reducing miR-143-3p bound to the *Npy* 3′ UTR. The relationship between miR-143-3p and *Npy* is particularly gripping given their involvement in metabolic pathways. Future studies are warranted to examine the connection between miR-143-3p and *Npy* under fasting and fed conditions and whether this relationship is disrupted in obese states. Dysregulated levels of miR-143 have been related to multiple disease contexts such as obesity [54], insulin resistance [54,55], and cardiovascular disorders [56,57]. For instance, morbidly obese adolescents and patients with metabolic syndrome have increased circulating miR-143 expression [58,59,60]. In HFD-fed mice, increased miR-143 expression in adipose tissues is correlated with higher body weight [61]. Moreover, whole-body deletion of miR-143 in HFD-fed mice lowers body weight and enhances energy expenditure, insulin sensitivity and glucose tolerance, and brown adipocytes thermogenesis [62]. Although the absence of miR-143 is associated with anti-obesity effects in the periphery, this role of miR-143 may be different at the central level. Since circulating miR-143 expression is elevated in obese states [58,59,60,61], this may increase the presence of miR-143 at the central level and decrease *Npy* mRNA, which would suppress feeding behaviour. However, this hypothesis requires further examination. 

Although the other candidate miRNAs did not directly bind the *Npy* 3′ UTR, there is substantial evidence that each of them may be involved in the regulation of energy homeostasis. Thus, it is plausible that they regulate the *Npy* gene indirectly. Perinatal HFD exposure increases miR-140-5p levels in adult ARC, and a poor metabolic environment in early life has been shown to impact long-term miRNA responses [63]. miR-140-5p has also been shown to promote oxidative stress in myocardial tissues by targeting *Nrf2*, a key regulator of antioxidative defense [47]. BPA induces oxidative stress that partially contributes to the differential changes in *Npy* expression [9]. In the mHypoE-41 cells, a BPA-mediated increase in miR-140-5p may therefore repress *Nrf2*, thereby aggravating the oxidative stress response and *Npy* dysregulation induced by BPA. 

The persistent downregulation of miR-29b-1-5p by BPA, regardless of hypothalamic models and timepoints, was particularly intriguing. miR-29b-1-5p originates from the miR-29 family, and members from this miRNA family have been linked to energy homeostasis through hypothalamic neurons. Ma et al. demonstrated that the absence of hypothalamic miR-29a/b-1 cluster in adult mice, which includes miR-29b-1-5p, led to the development of obesity [64]. This obesity-promoting effect is mediated through *Nras* that is targeted by miR-29a-3p and is a negative regulator of the PI3K-Akt-mTOR pathway [64]. Furthermore, obesogenic stimuli have been shown to dysregulate the expression of the miR-29 family. Depending on the tissue or cell type, HFD and saturated fatty acid exposure could either increase or decrease the levels of miR-29 family members [65,66,67,68]. 

NPY neurons have multifaceted roles in the hypothalamus, as they regulate reproductive function as well as energy homeostasis [40,41,42]. Feeding-related NPY neurons respond to anorexigenic signals, such as insulin, leptin, and estrogen by downregulating NPY expression [69,70,71]. NPY neurons also regulate the reproductive axis, and increased NPY secretion is crucial for preovulatory luteinizing hormone surge [72,73,74]. Population-specific effects of hormones on *Npy*-expressing hypothalamic cell lines have been described previously [9,42]. As an example, 17b-estradiol (E2) downregulates *Npy* in the mHypoE-42 neurons and upregulates *Npy* in the mHypoE-38 cells with a distinct temporal effect, and this differential regulation of *Npy* depends on the ratio of estrogen receptors [42]. As a result, mHypoE-42 cells are postulated to function as feeding-related NPY neurons considering their anorexigenic response to E2, while the mHypoE-38 cells may function as reproductive NPY neurons as the E2-mediated increase in NPY resembles the preovulatory response in vivo. Since miRNA candidates are differentially affected by BPA across *Npy*-expressing cell lines, this points towards the existence of population-specific effects of BPA on miRNAs. At the time and dose that were used in this study, BPA appears to interfere with individual miRNAs in neuronal subpopulations; however, BPA appears to have a minor impact on miRNA biogenesis components at the mRNA level in all the neuronal cell models.

Interestingly, BPA decreased *Npy* in the mHypoE-44 and mHypoE-46 cells [9]. Since BPA increased miR-143-3p levels in both cell lines, miR-143-3p may potentially contribute to decreased *Npy* in mHypoE-44 and mHypoE-46 cells by targeting the *Npy* 3′ UTR. Although the effect of BPA on miRNA does not fully correspond to the *Npy* mRNA changes across cell lines reported previously [9], the downstream impact of *Npy* may depend on the combination of miRNA changes induced by BPA. Therefore, studying individual miRNA in solitude may not yield insights into the cooperative action of miRNAs at the *Npy* 3′ UTR level. It is difficult to know whether *Npy* changes by BPA are mediated through multiple miRNAs acting at the *Npy* gene 3′ UTR, and future studies should consider delineating all miRNAs bound to this region through pull-down assays.

As observed in the miRNA mimic transfections, each miRNA mimic produces differential *Npy* changes between the mHypoE-41 and mHypoE-46 cells. Interestingly, *Npy* downregulation only occurred in the mHypoE-41 cells, while *Npy* upregulation was observed in mHypoE-46 cells. The dissimilarity of *Npy* responses to the same miRNA mimic between the cell lines infers that miRNA may have cell line-specific regulation of *Npy*. Heterogeneous NPY neuronal populations are present in vivo and in vitro [40,41,42], and each NPY subpopulation possesses distinct profiles of genes and miRNA expression [75]. mHypoE-41 and mHypoE-46 cells have been characterized previously and they represent distinct subpopulations of hypothalamic NPY neurons [9,76]. Hence, differences in the relative abundance of miRNAs and other genes, such as transcription factors and second messengers, between the cell lines would not only change the interaction between miRNAs but also the miRNA-mediated regulation of target genes. Since one miRNA can bind to many other target mRNAs, it can also indirectly regulate *Npy* through an intermediary. In other words, changes in *Npy* can occur without miRNAs acting on the *Npy* 3′ UTR directly as they can target other transcription factors or components of signaling pathways that ultimately influence *Npy* expression. 

Plausible transcription factor candidates could be ERα (*Esr1*) and ERβ (*Esr2*) because changes to the ERβ to ERα ratio can directly affect *Npy* expression in *Npy*-expressing cell lines [9,42]. It is noteworthy that a higher *Esr2*/*Esr1* ratio is associated with increased *Npy* expression in the mHypoE-46 cells. Hence, the different ER ratios between cell lines may contribute to the differential regulation of *Npy* mRNA by miRNAs in these cells. As demonstrated by Loganathan et al., ERβ is required for the BPA-induced decrease of *Npy* in the mHypoE-46 cells specifically. Blocking ERβ reversed the downregulation of *Npy* to an upregulation in the mHypoE-46 cells [9]. However, ERβ antagonism in another *Npy*-expressing cell line, mHypoA-59 cells, did not prevent or reverse BPA-induced changes of *Npy,* emphasizing cell-line specific regulation of *Npy* through ERβ activity in the mHypoE-46 cells [9]. Taking this into account, we speculate the contribution of miRNA candidates to ERβ antagonism via miRNA-mediated *Esr2* repression, which may prevent *Npy* downregulation in the mHypoE-46 cells. Since the ratio of ER subtypes is one determinant of *Npy* expression, miRNA-mediated changes in relative levels of *Esr2* to *Esr1* between mHypoE-46 and mHypoE-41 cells may contribute to the population-specific *Npy* phenotypes. *Esr1* and/or *Esr2* are predicted targets of miR-29b-1-5p, let-7b-3p, and let-7f-1-3p [31,32]. We found that miR-29b-1-5p and let-7f-1-3p mimics altered *Esr2* expression, while let-7b-3p mimics increased *Esr1* alone. Since ERs are involved in regulating numerous physiological pathways, it would be important to understand the potential implications of miR-29b-1-5p, let-7b-3p, and let-7f-1-3p in modulating ER-mediated signaling mechanisms, especially pathways contributing to overall energy homeostasis. 

## 5. Conclusions

This study aimed to delineate the involvement of hypothalamic miRNAs in regulating *Npy* mRNA expression. Our study uncovered a novel *Npy*-targeting miRNA, miR-143-3p, which targets the mouse *Npy* 3′ UTR in hypothalamic neurons. Although miR-140-5p, miR-29b-1-5p, and let-7b-3p alter *Npy* mRNA expression indirectly, each miRNA candidate has differential effects on *Npy*-expressing neurons, and miRNA-mediated regulation of *Npy* may vary depending on the ER subtype ratio, individual transcription factor, and signal transduction second messenger complement in each NPY neuronal subpopulation, as well as functional neuronal differences dictated by afferent neurons. The obesogenic BPA dysregulates miRNA expression in hypothalamic neuronal cell lines and primary cultures, highlighting the disruptive effects of BPA on hypothalamic neurons that are essential for energy balance. A growing body of literature highlights the involvement of miRNAs in a multitude of metabolic processes in the periphery. The regulation of energy homeostasis by hypothalamic miRNAs remains an underexplored area filled with exciting potential. Hence, the feasibility of adopting specific miRNAs as biomarkers or therapeutics for obesity warrants further exploration as more studies aim to understand the progression and development of metabolic diseases through expression profiling of miRNAs. Overall, the discovery of miR-143-3p as a miRNA that directly targets *Npy* highlights the potential of miRNA-mediated control of orexigenic neuropeptides to ultimately influence the hypothalamic control of energy homeostasis. 

## Figures and Tables

**Table 1:** Primer sequences for RT-qPCR.

Gene or miRNA	Forward Primer Sequence (5′ to 3′)	Reverse Primer Sequence (5′ to 3′)
*Rpl7*	TCGCAGAGTTGAAGGTGAAG	GCCTGTACTCCTTGTGATAGTG
*Npy*	CAGAAAACGCCCCCAGAA	AAAAGTCGGGAGAACAAGTTTCATT
*Dicer*	TCGAGCCTCCATTGTTGGTC	TGGTCTCCTCCTCGTCATGT
*Drosha*	CCCGGAGAAGAGGCAATCAA	GGTCAGAGGAGCATGTGCAA
*Dgcr8*	TGCAAAGATGAATCAGTTGATCTGG	TTCCGCTTCATCTCACGGTT
*Ago2*	TTCCCACTACCACGTGCTTT	GCTTCCTTCAGCGCTGTCAT
*Esr1*	GAGTGCCAGGCTTTGGGGACTT	CCATGGAGCGCCAGACGAGA
*Esr2*	ATCTGTCCAGCCACGAATCAGTGT	TCTCCTGGATCCACACTTGACCAT
*Nrf2*	GGACATGGAGCAAGTTTGGC	CCAGCGAGGAGATCGATGAG
*Bmal1*	GGGAGGCCCACAGTCAGATT	GTACCAAAGAAGCCAATTCATCAA
Mmu-miR-221-3p	GCAGAGCTACATTGTCTGCT	CAGTTTTTTTTTTTTTTTGAAACCCA
Mmu-miR-143-3p	CAGTGAGATGAAGCACTGTAG	GGTCCAGTTTTTTTTTTTTTTTGAG
Mmu-miR-140-5p	CAGCAGTGGTTTTACCCTATG	GGTCCAGTTTTTTTTTTTTTTTCTAC
Mmu-let-7f-1-3p	GCAGCTATACAATCTATTGCCT	GTCCAGTTTTTTTTTTTTTTTGGGA
Mmu-let-7b-3p	CAGCTATACAACCTACTGCCT	GTCCAGTTTTTTTTTTTTTTTGGGA
Mmu-miR-29b-1-5p	GCAGGCTGGTTTCATATGG	TCCAGTTTTTTTTTTTTTTTAAACCAC

**Table 2:** Putative *Npy*-targeting miRNAs based on TargetScan prediction.

miRNA	Binding Position in the *Npy* 3′ UTR	Expression Confidence	Annotation Confidence
mmu-miR-147-5p	35–41	Low	High
**mmu-miR-143-3p**	45–52, 50–57	High	High
mmu-miR-29b-2-5p	64–70	High	High
**mmu-miR-29b-1-5p**	65–71	High	High
mmu-miR-698-5p	76–87	High	High
mmu-miR-92a-2-5p	81–87	High	High
mmu-miR-33-5p	86–92	High	High
**mmu-miR-140-5p**	97–103	High	High
mmu-miR-25-5p	111–117	High	High
mmu-let-7a-1-3p	139–145	Low	High
mmu-let-7c-2-3p	139–145	Low	High
**mmu-let-7b-3p**	139–145	High	High
mmu-miR-98-3p	139–145	Low	High
**mmu-let-7f-1-3p**	139–145	High	High
mmu-miR-381-3p	139–145	High	High
mmu-miR-539-3p	139–145	Low	High
mmu-miR-669b-3p	140–147	Low	High
mmu-miR-669f-3p	140–147	High	High
mmu-miR-669c-3p	148–154	High	High

5 putative *Npy*-targeting miRNAs (in bold) fulfilled selection criteria from in silico analysis and in vitro validation. Expression confidence was determined with hypothalamic miRNA array data. miRNAs with detection above background (DABG) values (*p* < 0.05) in the whole hypothalamus or immortalized hypothalamic neurons were considered as high expression confidence. Annotation confidence was determined by miRbase, and bona fide miRNAs were considered as ‘high confidence’.

**Table 3:** Summary of *Npy* and *Esr2/Esr1* regulation by miRNA mimics in the mHypoE-41 and mHypoE-46 cells.

	mHypoE-41		mHypoE-46	
miRNA Mimic	*Npy* Expression	*Esr2/Esr1*	*Npy* Expression	*Esr2/Esr1*
miR-143-3p	↓	1.02	~	2.16
miR-140-5p	↓	0.92	↑	1.70
miR-29b-1-5p	~	0.99	↑	1.68
let-7b-3p	↓	0.96	~	0.49
let-7f-1-3p	~	0.85	~	0.87

The direction of *Npy* change is denoted by ↓ (down), ↑ (up), ~ (no change). The magnitude change in *Esr2* over *Esr1* with miRNA mimics is represented by a numerical value.

## Data Availability

The data presented in this study are available on request from the corresponding author.
